# Can the Macrogeometry of Dental Implants Influence Guided Bone Regeneration in Buccal Bone Defects? Histomorphometric and Biomechanical Analysis in Beagle Dogs

**DOI:** 10.3390/jcm8050618

**Published:** 2019-05-07

**Authors:** Manuel Fernández-Domínguez, Victor Ortega-Asensio, Elena Fuentes Numancia, Juan Manuel Aragoneses, Horia Mihail Barbu, María Piedad Ramírez-Fernández, Rafael Arcesio Delgado-Ruiz, José Luis Calvo-Guirado, Nahum Samet, Sergio Alexandre Gehrke

**Affiliations:** 1 Maxillofacial Department HM Hospitals, Doctoral Program of Translational Medicine, CEU San Pablo University, 28223 Madrid, Spain; clinferfun@yahoo.es; 2 Department of Implant Dentistry, CEU San Pablo University, 28223 Madrid, Spain; v.ortegasensio@gmail.com (V.O.-A.); efuentesnumancia@gmail.com (E.F.N.); 3 Department of Dental Research, Universidad Federico Henriquez y Carvajal (UFHEC), Santo Domingo 10107, Dominican Republic; jaragoneses@ufhec.edu.do; 4 Faculty of Dental Medicine, University Tito Maiorescu, 004051 Bucarest, Romania; horia.barbu@prof.utm.ro; 5 Department of Oral and Implant Surgery, Faculty of Health Sciences, Universidad Católica San Antonio de Murcia (UCAM), 30107 Murcia, Spain; mpramirez@ucam.edu; 6 Department of Prosthodontics and Digital Technology, Stony Brook University, Stony Brook, New York, NY 11794-8712, USA; Rafael.Delgado-Ruiz@stonybrookmedicine.edu; 7 Faculty of Health Sciences, Universidad Católica San Antonio de Murcia (UCAM), 30107 Murcia, Spain; 8 Private practice. Tel Aviv 3100000, Israel; samet@drsamet.com; 9 Department of Research, Biotecnos, Cuareim 1483, Montevideo CP 11100, Uruguay; sergio.gehrke@hotmail.com

**Keywords:** dental implants, guided bone regeneration, buccal defects, implant macrogeometry, implant stability quotient.

## Abstract

The aim of this experimental animal study was to assess guided bone regeneration (GBR) and implant stability (ISQ) around two dental implants with different macrogeometries. Forty eight dental implants were placed within six Beagle dogs. The implants were divided into two groups (*n* = 24 per group): G1 group implants presented semi-conical macrogeometry, a low apical self-tapping portion, and an external hexagonal connection (whereby the cervical portion was bigger than the implant body). G2 group implants presented parallel walls macrogeometry, a strong apical self-tapping portion, and an external hexagonal connection (with the cervical portion parallel to the implant body). Buccal (mouth-related) defects of 2 mm (c2 condition) and 5 mm (c3 condition) were created. For the control condition with no defect (c1), implants were installed at crestal bone level. Eight implants in each group were installed under each condition. The implant stability quotient (ISQ) was measured immediately after implant placement, and on the day of sacrifice (3 months after the implant placement). Histological and histomorphometric procedures and analysis were performed to assess all samples, measuring crestal bone loss (CBL) and bone-to-implant contact (BIC). The data obtained were compared with statistical significance set at *p* < 0.05. The ISQ results showed a similar evolution between the groups at the two evaluation times, although higher values were found in the G1 group under all conditions. Within the limitations of this animal study, it may be concluded that implant macrogeometry is an important factor influencing guided bone regeneration in buccal defects. Group G1 showed better buccal bone regeneration (CBL) and BIC % at 3 months follow up, also parallel collar design can stimulate bone regeneration more than divergent collar design implants. The apical portion of the implant, with a stronger self-tapping feature, may provide better initial stability, even in the presence of a bone defect in the buccal area.

## 1. Introduction

The quantity of peri-implant tissues resulting from remodeling over a 12-week period is influenced by several important factors: Placement in fresh extraction sockets or at healed sites; immediate or delayed placement following extraction or dental loss [1,2].

Maxillary bone atrophy generally causes dehiscence at the moment of implant placement surgery. The potential for successful regeneration depends upon the dimensions of the dehiscence, and it is essential to consider this parameter in order to achieve adequate regeneration outcomes [3].

Most synthetic fillers available on the market are based on hydroxyapatite and tricalcium phosphate derivatives. These substitutes may vary in their chemical characteristics, i.e., elasticity, grain size, and texture. Although these materials have a lower regenerative capacity in comparison with autologous bone (which provides progenitor bone cells with a perfect scaffold for growth and regeneration), they do obtain adequate and predictable outcomes [2,4].

Regarding implant macrogeometry, several design variations have been proposed, including conical or cylindrical forms, the presence of cervical microthreads, polishing or other surface treatments, all of which obtain different results [5,6]. Surfaces modified with different acids or H_2_O_2_ have been found to produce similar osseointegration compared to a standard sandblasted and acid-etched surface. For bicortically installed implants, the portion in close contact with cortical bone presents a higher percentage of osseointegration compared with the implant portion in contact with the bone marrow [7]. Implant diameter has not been found to affect crestal bone loss, but BIC values are affected by the implant’s diameter and design, whereby narrow implants obtain higher BIC values than mini-implants [8]. Nevertheless, sub-crestal placement of either implant type favors crestal bone preservation, while crestal placement of either design is associated with crestal bone loss. It has also been shown that narrow implants protect peri-implant crestal bone [8,9,10,11].

Immediately following implant placement, the stability obtained in the first instance is known as primary stability. But stability decreases as healing proceeds, and is later replaced by what is called secondary stability, which places a real value on the success of the implant’s osteointegration in the long term. The Osstell^®^ system provides a good means of evaluating implant stability. Its use was first reported by Meredith et al. [12], who described it as a non-invasive technique based on resonance frequency analysis (RFA). The portable device measures resonance, from which it calculates an implant stability quotient (ISQ) [13]. Histomorphometric analysis provides a means of observing peri-implant bone and its contact with the implant. The main parameters evaluated in histomorphometric analysis are bone-to-implant contact (BIC), which describes the quantity of bone surface in direct contact with the implant surface, and crestal bone loss (CBL), which describes the situation of the crestal bone by measuring the distance from the implant shoulder to the first point of BIC [14]. In cases of atrophic maxilla, it is not clear which factors should be considered when selecting an implant design and filling material, and so there is a pressing need to define the parameters and considerations that would help to make the right choices and maximize implant success rates [15]. In this context, the present experimental study aimed to assess the regenerative behavior (based on ISQ values, BIC and CBL) of two different implants placed with buccal defects of 2 and 5 mm, comparing two different macrogeometries, and monitoring their interaction with guided bone regeneration using a particle bone graft material. 

## 2. Experimental Section

### 2.1. Materials and Methods

#### 2.1.1. Animal Preparation and Care

Six Beagle dogs, aged 2–5 years and weighing between 14 to 15 kg, were used in this experimental study. The Beagle dogs were supplied by the animal unit at Gomez Ulla Military Hospital, Madrid, Spain. The animals’ housing and care were approved by the hospital’s Institutional Animal Care and Use Committee (Report Nº 006 16-04-2015, Comité de ética de Experimentación animal del Centro Militar de Veterinaria de la Defensa, Madrid (CEMILVETDEF) and followed European Union guidelines for minimizing animal pain, distress, and suffering. 

#### 2.1.2. Materials and Group Formation

Two dental implants with different macro geometries and similar microgeometry (surface treatment) were used, forming the following groups: Group 1 (G1 group): Dental implants made of grade 5 titanium alloy (Ti-6Al-4V - Titanium/Aluminum/Vanadium); the macroscopic design had parallel walls in the middle third, but was conical in the apical and coronal thirds, with a small self-tapping apex; surface treatment consisted of blasting with aluminum oxide (Al_2_O_3_) particles (size ~300 microns), followed by etching with hydrochloric acid, creating Ra 1.3 ± 0.17 roughness on the implant surface. All implants used were 3.3 mm in diameter by 10 mm in length, with an external hexagon of 0.7 mm height, and 2.4 mm diameter, and a thread pitch of 2 mm (Klockner KL external hex; Klockner SA, Barcelona, Spain). 

Group 2 (G2 group): dental implants made of grade 5 titanium. The macroscopic design had parallel walls along half the implant, and then a high self-tapping design at the apex, designed to increase primary stability; surface treatment consisted of blasting with Al_2_O_3_ particles (size ~300–400 microns) followed by etching with hydrochloric/sulfuric acid, creating Ra 1.25 + 0.16 roughness. All implants were 3.3 mm in diameter by 10 mm in length, with an external hexagon of 0.7 mm height and 2.45 mm diameter, and a thread pitch of 0.8 mm (Biocom external hex; MIS Implants, Bar-Lev Industrial Zone, Israel).

**Figure 1:**
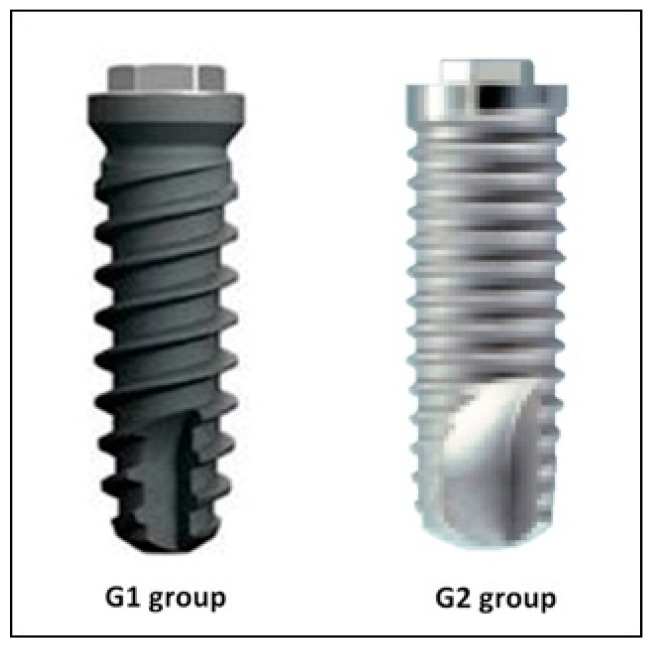
Images of the implants assayed, comparing guided bone regeneration in induced buccal wall defects. Both implants had similar microgeometry.

Forty-eight implants (*n* = 24 per group) were placed into the six animals. The two implant designs are shown in Figure 1.

Guided bone regeneration was performed to fill buccal defects, using the particle bone graft material Max Resorb^®^ (Botiss Biomaterials GmbH, Zossen, Germany), a bone replacement material composed of beta-tricalcium phosphate (40%) and hydroxyapatite (60%), which is fully synthetic, and has a granule size of 0.8–1.5 mm. A resorbable membrane Jason Membrane^®^ (Botiss Biomaterials GmbH, Zossen, Germany) was used to cover the graft material.

### 2.2. Surgical Procedures

For all surgical procedures, anesthesia included premedication with intramuscular acepromazine (maximum 0.1 mg/kg) and atropine sulfate 0.5 mg. The animals were sedated with ketamine (5–8 mg/kg intravenously), and a mix of isoflurane (1.5–2.0%) and N_2_Or: Or_2_ (1:1) administered by the endotracheal route. Antibiotic treatment comprised intramuscular administration of Baytril 10% (1.5 mL/day) and Borgal 24% (3 mL/day) administered 24 h before surgery, and after the surgical procedures for two further weeks. The animals were fed a soft diet for one month after surgery. The sites corresponding to the premolars teeth (P1, P2, P3, and P4) in both hemiarches of the mandible were prepared 90 days before implant placement, extracting the teeth, and leaving the alveolae to heal. 

After 90 days’ healing, the anesthesia procedures and surgical care were repeated. A crestal incision and flap elevation were performed from a point distal of the canine to mesial of the first molar.

**Figure 2:**
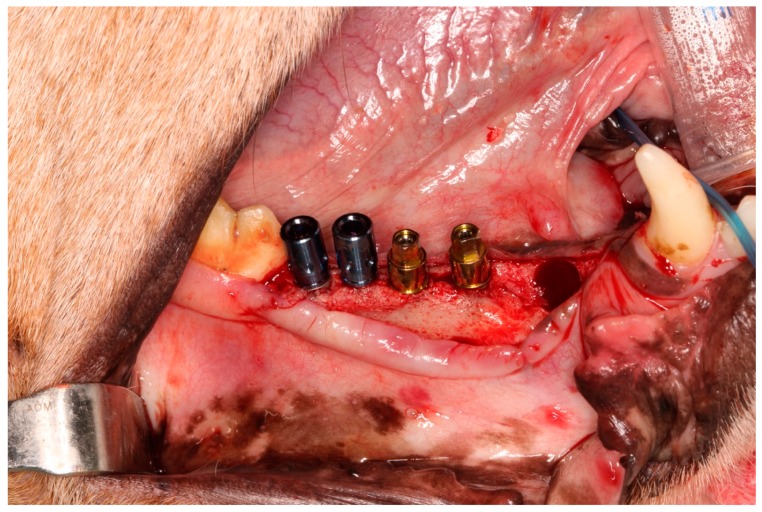
Four implants of two different types, placed in each dog´s hemimandible.

Four implants were placed (two Group G1 implants and two for Group G2) in each hemimandible (Figure 2), following procedures recommended by the manufacturer of each implant system. All implants were inserted applying 35 Ncm torque. 

After implant placement three conditions were created (*n* = 8 per condition): 

Condition 1 (c1) used as a control sample; the implants were placed at crestal bone level.

Condition 2 (c2); a defect of 2 mm was created on the buccal bone wall.

Condition 3 (c3); a defect of 5 mm was created on the buccal bone wall. 

**Figure 3:**
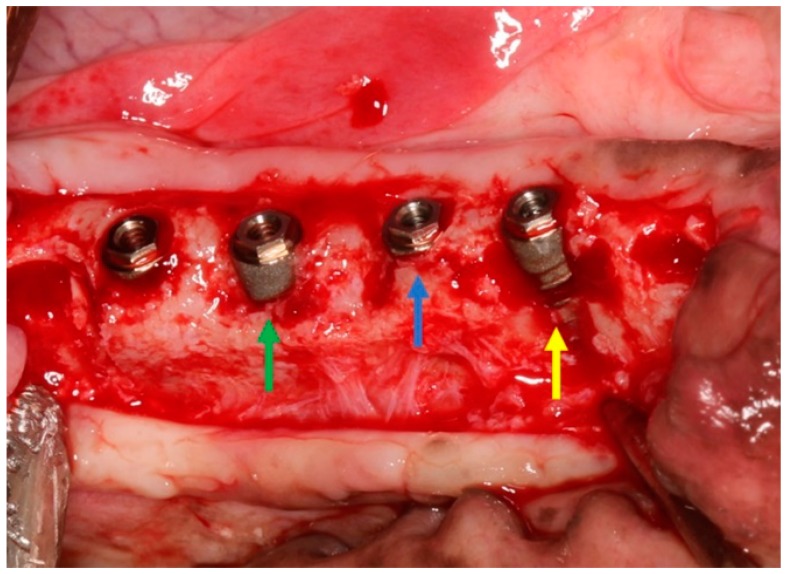
The clinical image shows the three conditions applied in the experiment: control site (blue arrow), induced buccal defect of 2 mm (green arrow), and 5 mm defect (yellow arrow).

To create the buccal defects, a round tungsten bur was used to eliminate buccal bone. Figure 3 shows the three conditions applied in the experiment. All implants and conditions were distributed randomly using the website http://www.randomization.org (Version 4.0, September 2015). 

Immediately after implant installation (time 1), a smart peg was placed on each implant and ISQ values were measured using the Osstell^®^ device (Integration Diagnostics Ltd., Goteborgsvagen, Sweden).

**Figure 4:**
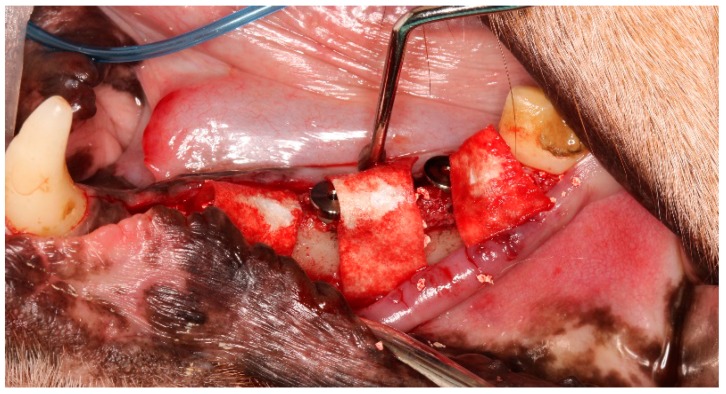
Guided bone regeneration (bone graft and membrane) of the induced 2 mm and 5 mm defects.

Then, the defects were filled with bone graft material and covered with a resorbable membrane. The wounds were closed with resorbable sutures using Vicryl 4-0 (Figure 4).

Three months after implant healing, the ISQ values were measured again (time 2), the animals were sacrificed, and the samples extracted for histological and histomorphometric study. The ISQ values were measured in two directions, buccal to lingual and lingual to buccal, calculating a mean and standard deviation for each implant.

### 2.3. Histological Procedures and Preparation

To remove the samples, an oscillating saw (Dentalcare, Cox, Alicante, Spain) was used with manual irrigation. The samples were submerged in 10% formaldehyde solution for one week at a temperature of 4 °C. Biopsies were processed using the method described by Donath and Breuner [16]. Samples were dehydrated with an ascending ethanol series and infiltrated in Technovit 7.200 VCL- resin (Kulzer, Friedrichsdorf, Germany). Then the samples were reduced to a thickness of 50 μm by micro grinding, followed by polishing with an abrasive system. Afterwards, they were stained with picro-syrius-hematoxylin [17,18].

For histomorphometric analysis of the bone defects, images of the histological sections were captured with a Leica^®^ DFC425 digital camera linked to a Leica DM6000 microscope (using polarized light) connected to an Hp DVD 1260 computer. Histomorphometric measurements were made using Leica MMAF 1.4 software (MetaMorph, Leica, Wetzlar, Germany).

**Figure 5:**
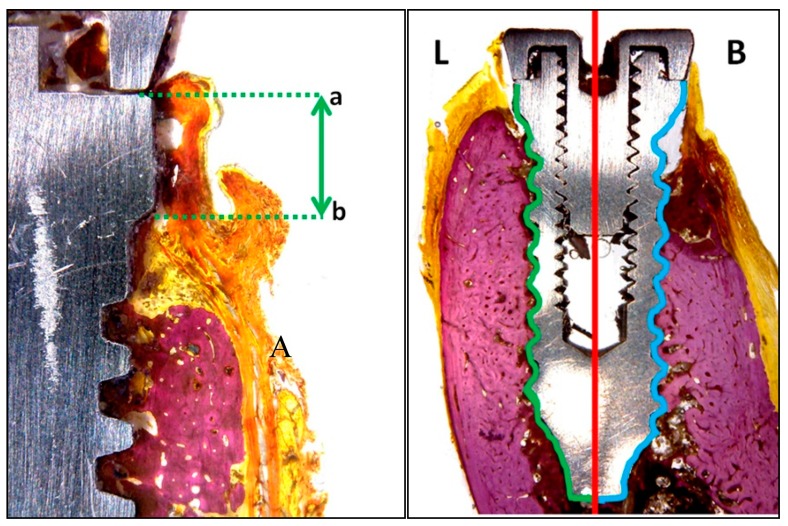
Schematic histological images showing measurement parameters: (**A**) crestal bone loss (CBL); (a) implant shoulder to (b) first point of bone contact. (**B**) Bone-to-implant contact (BIC)% measurement; in the lingual portion (L) the green line was considered, and in the buccal portion (B), the blue line was considered.

Histological analysis evaluated the peri-implant tissues as follows (Figure 5):

- Vestibular and lingual crestal bone loss (CBL) quantified the amount of bone loss (or bone that had failed to regenerate) around implants placed at sites with or without defects, measuring from the implant platform to the first point of bone-to-implant contact (Figure 5a).
- Bone-to-implant contact (BIC %) registered the percentage of bone in direct contact with the implant surface. To measure this parameter, the implant was divided at the center into two parts: the lingual portion and buccal portion, both extending from the implant shoulder to the apical portion (Figure 5b).

### 2.4. Statistical Analysis

The non-parametric Kruskal Wallis test was used to analyze the data obtained. It was not possible to use a parametric test, as this would require implant numbers greater than 30, and furthermore, the samples did not fulfill normality of variance or homogeneity. To compare BIC and CBL between group G1 and group G2, the Mann-Whitney U test was applied. Statistical significance was set at *p* < 0.05. 

## 3. Results

All animals showed good postoperative evolution, free from abnormal inflammation or healing problems. Three months after implant placement, all implants presented adequate osseointegration. ISQ values under the three conditions applied show differences between the two implant design groups. At time 1 (immediately after implant placement) group G1 samples showed a significant difference between the conditions (*p* = 0.0224). At time 2 (3 months after implant placement) comparison of mean and standard deviation ISQ values showed significant differences (*t*-Test) between the two groups under each condition. (Table 1).

Regarding lingual and buccal CBL measurements, the G1 group presented better behavior in comparison to the G2 group under all conditions on both implant sides. Data for both groups under each condition are shown in Table 2.

The same happened with BIC values (%), which were higher in group G1 (Klockner implant) than group G2 (MIS implant), which was to be expected in light of the lower crestal bone loss obtained in group G1. 

CBL values obtained in both groups showed significant differences between the three conditions within each group (*p* < 0.0001). Data (mean, standard deviation and statistical comparison) are summarized in Table 3, showing statistical differences for practically all conditions when compared between groups. CBL was lower in group G1 under all conditions.

In general, BIC% showed statistical differences between the groups under all conditions, and within both groups under all conditions. Table 4 and Table 5 show mean, standard deviation, median and statistical comparison between groups and conditions.

CBL values obtained and analyzed with the Kruskal-Wallis test to compare control with regenerated implants did not present statistically significant differences (*p* < 0.05) with the exception of GBR in 5 mm defects, which was better for Klockner implants (Table 6).

Implants with 2 mm regenerated defects presented worse vestibular and lingual CBL values than control implants: This was also true for mean % BIC. Implants with 5 mm defects showed worse CBL values than control implants, but similar to the implants with 2 mm defects on both vestibular and lingual aspects, while 5 mm defects showed the lowest mean BIC%.

## 4. Discussion

The present experimental animal study set out to assess the regenerative behavior (based on ISQ values, BIC and CBL) of implants placed with buccal defects of 2 and 5 mm, comparing two different macrogeometries in relation to guided bone regeneration using a particle bone graft material. The clinical situations tested (buccal defects of different sizes, guided bone regeneration) were selected because they are very common in the practice of implant dentistry (loss of buccal bone), in which the use of GBR procedures involving bone graft materials plus membrane, is an adequate and commonly applied option [19,20]. In the literature, there are several instances of clinical evidence that demonstrates that the survival rates of implants placed simultaneously to GBR are similar to the results of implants inserted into native bone [21,22,23,24,25]. But despite the evidence in favor of GBR, few studies have investigated the role played by implant macrogeometry in bone regeneration using this procedure. According to the present results, in both implant groups, condition c1 (control samples without buccal defect) obtained a better histological (BIC and CBL) and ISQ values, when compared with implants placed with buccal defects (conditions c2 and c3). At the same time, when the two implant macrogeometries were compared, the group G1 implants showed better performance in comparison with group G2.

Several studies have demonstrated the influence of implant macrogeometry on the behavior of the peri-implant tissues and crestal bone healing. Implant neck configurations can directly influence marginal bone loss. Previous studies have reported a strong association between crestal bone behavior and remodeling after the placement of implants with different cervical designs [26,27]. In this way, the present study compared healing processes using GBR in buccal defects around implants with an expanded cervical portion (group G1), and implants with a parallel cervical portion (group G2). Histological evaluation obtained less CBL in group G1 samples under all conditions. 

Buccal CBL can be considered the most important variable for assessing the success or failure of GBR by means of biomaterials and membranes. The present study investigated induced defects of two sizes: 2 mm defects considered ‘small,’ and 5 mm defects considered ‘large’ in order to compare this variable. Differences were found between the 2 mm and 5 mm defects. This could be due to the triangular shape of the Beagle jaw – similar to the human mandible – and is the most complex area when it comes to regeneration, because it involves the first 2 mm of the implant in an area where the implant is wider, but the mesial and distal bone walls are thinner. When the vestibular wall (below this 2 mm) is thicker, the defect will regenerate completely [20]. Regarding implant macrogeometry, the histomorphometric measurements obtained in the present study confirmed that cervical implant design influences changes to the alveolar ridge (crestal bone) following implant placement, regardless of the regenerative procedures applied. Analysis was performed three months after implant placement because other animal studies have concluded that the marginal bone gaps present at the time of implant placement fill completely within three months [28,29,30,31]. 

The increased BIC values for implants with a higher self-tapping conical apical design (Group 1) and parallel walls design (group G2) suggest that bone regeneration with this design may be more favorable in comparison with the macrogeometry presented by group G2 parallel walls implants. These results concur with previous findings in an animal study, which suggested that the implant collar configuration may favor crestal bone preservation [32,33,34]. Comparing BIC values obtained by MIS and Klockner implants, Klockner obtained higher values than MIS implants for control samples and for samples with 5 mm defects; with 2 mm defects, both implants presented similar outcomes.

Finally, some studies have demonstrated that higher ISQ values do not necessarily mean that implants present better osteointegration [35], but it is accepted that an implant presenting an ISQ value over 50 may be loaded safely [35,36]. ISQ values can oscillate between 0 and 100 (measurements of between 3500 and 8500 Hz). The ISQ values obtained with group G1 implants showed a similar evolution between the two times analyzed to group G2. The ISQ for condition c1 (without defect) showed higher values in both groups in comparison with conditions c2 and c3 (defects of 2 mm and 5 m, respectively). However, the larger buccal defect (condition c3) showed an important decrease in ISQ in group G1, but the decrease was not so critical in the G2 group samples. This was probably a consequence of the differences in implant design, since the stability of the group G2 design is based in the implant root, while with group G1 design, stability derives from the apical design (with a higher self-tapping feature). These results were similar to the study published by Gehrke and Marin (2015), which compared the stability of implants with different apical designs [37]. In this way, other clinical studies have reported that small dehiscence defects left to heal spontaneously do not have an effect on implant stability [38,39,40]. But other authors, evaluating stability after a 12-week healing period, report more favorable implant stability with guided bone regeneration [41]. Nevertheless, ISQ readings do not always correlate to the histological values obtained, so in research situations, histological analysis is necessary to obtain a reliable assessment of osteointegration. It should be noted that when the distance between turns is taken into consideration, the Klockner implant has more distance between each (2 mm) than the MIS implant (1.8 mm), which should mean less stability, but as we have already mentioned, the main anchoring force of this Klockner implant is at its apex, and this makes the distance found between each loop irrelevant for ISQ values. The limitations of the study are to transpose the data of the implants placed in dogs to the humans. The buccal bone wall of the dog is normally similar to that of the human, therefore the placement of the implants practically behaves the same. In the current study the difference has been the creation of a buccal defect with which the parallel platform implants are the most indicated when a patient presents buccal lesions, compared with implants with an expanded platform which are not indicated in those cases.

## 5. Conclusions

Within the limitations of this animal study may be concluded that implant macrogeometry is an important factor to consider in relation to guided bone regeneration in buccal defects. Implants with a conical design (Klockner implants) and smooth collar may promote bone regeneration more effectively in comparison with implants with parallel walls design on crestal areas. The apical portion of the implant with a higher self-tapping feature may improve initial stability even in the presence of a bone defect in the buccal area.

## Figures and Tables

**Table 1:** The comparison of mean, standard deviation values and statistical differences of the values of the two groups in each conditions and different times.

	Time 1			Time 2		
Group	c1	c2	c3	c1	c2	c3
G1	75.3 ± 5.10 ^a^	73.9 ± 4.35	71.6 ± 4.06 ^a,b^	81.2 ± 3.79 ^a^	78.5 ± 4.92	77.5 ± 5.32 ^a,b^
G2	73.9 ± 2.38 ^a,b^	71.9 ± 4.38	67.8 ± 5.06 ^a,b^	78.8 ± 1.69 ^a,b^	75.7 ± 3.45 ^a^	72.6 ± 3.01 ^b^

(a) statistical difference between the conditions in the same group and time (*p* < 0.05). (b) statistical difference between the groups in the same time and condition (*p* < 0.05).

**Table 2:** Mean crestal bone loss values were lower in group G1 (Klockner implants) compared with group G2 (MIS implants) on both vestibular and lingual aspects.

		*N*	Mean	SD	Mean + Standard Error
Crestal Bone Loss (CBL) Buccal	MIS Biocom	29	2.72293	1.905044	0.353758
	Klockner	26	1.80527	1.193518	0.234068
CBL Lingual	MIS Biocom	29	1.90383	1.72385	0.320111
	Klockner	26	1.65369	1.631999	0.320061
BIC %	MIS Biocom	29	79.559	12.99686	2.41346
	Klockner	26	86.5354	7.9248	1.55418

**Table 3:** Height from implant shoulder to first point of bone-implant contact (CBL): Mean, standard deviation and data comparison (*t*-test). * Statistically significant difference (*p* < 0.05).

CBL	LINGUAL			BUCCAL		
Group	c1	c2	c3	c1	c2	c3
	mean ± SD	mean ± SD	mean ± SD	mean ± SD	mean ± SD	mean ± SD
G1	0.3 ± 0.21	0.5 ± 0.22	0.7 ± 0.16	0.3 ± 0.21	0.6 ± 0.18	1.4 ± 0.25
G2	0.4 ± 0.19	0.7 ± 0.17	1.0 ± 0.25	0.9 ± 0.17	1.1 ± 0.23	2.3 ± 0.23
*p*-value	0.1971	0.0195 *	0.0164 *	0.0009 *	0.0031 *	0.0009 *

**Table 4:** Comparison of lingual BIC% values between the two groups under the three conditions at time 2 (3 months after implant placement).

	Group G1		Group G2	
Condition	Mean ± SD	Median	Mean ± SD	Median
c1	73.25 ± 3.12 ^b^	73.32	70.52 ± 4.03 ^b^	70.75
c2	72.97 ± 4.11 ^b^	73.05	69.62 ± 4.43 ^b^	69.88
c3	71.43 ± 3.98 ^b^	71.42	68.32 ± 4.66 ^b^	68.55

Data include mean, SD and medians. Significant differences (*p* < 0.05): (a) Comparison between conditions within the same group; (b) Comparison of the same conditions between groups.

**Table 5:** Comparison of buccal BIC % values between the two groups under the three conditions at time 2 (3 months after implant placement).

	G1 Group		G2 Group	
Condition	Mean ± SD	Median	Mean ± SD	Median
c1	73.98 ± 3.65 ^a,b^	74.03	70.43 ± 3.65 ^a,b^	70.50
c2	71.04 ± 4.49 ^a,b^	71.10	65.23 ± 4.11 ^a,b^	65.37
c3	68.40 ± 4.08 ^a,b^	69.00	60.72 ± 4.29 ^a,b^	60.85

Data include mean, SD and medians. Significant differences (*p* < 0.05): (a) comparison between conditions within the same group; (b) Comparison of the same conditions between groups.

**Table 6:** Comparison of mean vestibular crestal bone loss (CBL) between three conditions and the two implant groups at Time 2 (3 months after implant placement).

CBL Vestibular	Implant	Mean ± SD	*p*-Value
No treatment	MIS	2.40 ± 0.11	0.671
	Klockner	1.59 ± 0.22 *	0.128 *
	Total	1.99 ± 0.34	0.529
guided bone regeneration (GBR) 2 mm	MIS	2.68 ± 0.28	0.762
	Klockner	2.16 ± 0.17	0.821
	Total	2.42± 0.23	0.651
GBR 5 mm	MIS	3.03± 0.53	0.566
	Klockner	1.90± 0.38 *	0.321 *
	Total	2.47± 0.35	0.762

Mean and standard deviation. Significant differences (* *p* < 0.05).
